# 286. The Effect of Fecal Microbiota Transplantation on Decolonization of Multidrug-resistant Organisms in the gut

**DOI:** 10.1093/ofid/ofad500.358

**Published:** 2023-11-27

**Authors:** Seung Soon Lee, Ki Tae Suk, Dong eun Yong

**Affiliations:** Hallym University Chuncheon Sacred Heart Hospital, Chuncheon, Kangwon-do, Republic of Korea; Chuncheon Sacred Heart Hospital, Hallym University College of Medicine, Chuncheon-si, Kangwon-do, Republic of Korea; Yonsei University College of Medicine, Seoul, Seoul-t'ukpyolsi, Republic of Korea

## Abstract

**Background:**

Gut colonization of multidrug-resistant organisms (MDROs), such as carbapenemase-producing Enterobacteriaceae (CPE) and vancomycin-resistant Enterococci (VRE), precedes clinical infections. It increases the risk of patient-to-patient transmission of MDROs as well as clinical infections such as bacteremia. Therefore, decolonization of MDROs in the gut by restoring colonization resistance through fecal microbiota transplantation (FMT) could be an effective infection control strategy for MDROs.

**Methods:**

From March 2019 to November 2022, FMT for CPE and VRE decolonization in the gut was conducted at Hallym University Chuncheon Sacred Heart Hospital. Frozen or capsulized stool (MicroBiotix FMT Delivery Microbiota Preparation) from unrelated pre-screened healthy donor was used for each FMT. The rates of and the time to successful decolonization, defined as 3 consecutive negative results from rectal swab culture repeated with 3-day intervals after the initial FMT, were investigated in CPE and VRE rectal colonizers, respectively.

**Results:**

FMT was performed in 60 CPE rectal carriers (KPC=55, KPC & NDM=2, KPC & IMP=1, NDM=1, NDM & OXA=1) and in 15 VRE rectal carriers (*E. faecium*=14, *E. faecalis*=1). Among patients with follow-up data after initial FMT, decolonization success rates for CPE and VRE rectal carriers were 44.8% (26/58) and 66.7% (10/15) at 1 month, 77.1% (37/48) and 92.9% (13/14) at 3 months, 95.5% (42/44) and 100% (14/14) at 6 months, respectively. Median and mean times to decolonization after initial FMT were 30 and 43 days in CPE rectal colonizers and 14 and 29 days in VRE rectal colonizers, respectively.

FMT results for decolonization of MDROs in the gut of Chuncheon Sacred Heart Hospital (ClinicalTrials.gov number, NCT04583098)

**Figure:**
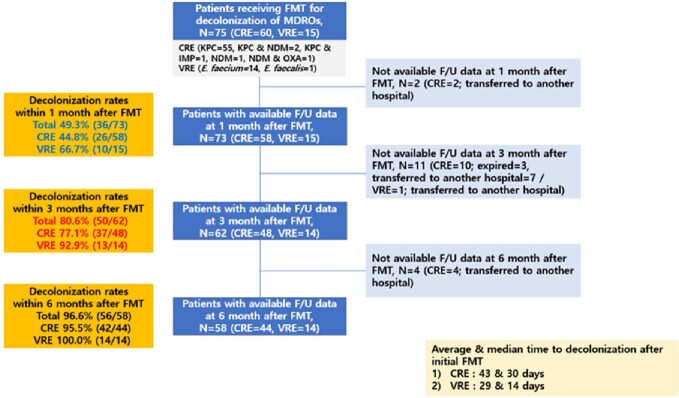

**Conclusion:**

FMT as a microbiome restoration therapy is an effective method to control CPE and VRE colonization (ClinicalTrials.gov number, NCT04583098).

**Disclosures:**

**All Authors**: No reported disclosures

